# The Combination Empagliflozin/Metformin Attenuates the Progression of Metabolic Dysfunction-Associated Steatotic Liver Disease in a Diet-Induced Experimental Rat Model

**DOI:** 10.3390/ijms26189010

**Published:** 2025-09-16

**Authors:** Oscar René Zambrano-Vásquez, Fernando Cortes-Camacho, Juan Carlos Cabrera-Angeles, Ana Lilia Hernández-Alba, Fernando Enrique García-Arroyo, Jorge Ismael Castañeda-Sánchez, Elena Aréchaga-Ocampo, Omar Emiliano Aparicio-Trejo, Leonardo Del Valle-Mondragón, Constanza Estefanía Martínez-Olivares, Rogelio Hernández-Pando, Laura Gabriela Sánchez-Lozada, Horacio Osorio-Alonso

**Affiliations:** 1 Doctorado en Ciencias Biológicas y de la Salud, Universidad Autónoma Metropolitana, Mexico City 14387, Mexico; oscar.zambrano@cua.uam.mx (O.R.Z.-V.); fernando.cortes@cua.uam.mx (F.C.-C.); 2 Departamento de Fisiopatología Cardio-Renal, Instituto Nacional de Cardiología Ignacio Chávez, Mexico City 14080, Mexico; enrique.garcia@cardiologia.org.mx (F.E.G.-A.); omar.aparicio@cardiologia.org.mx (O.E.A.-T.); laura.sanchez@cardiologia.org.mx (L.G.S.-L.); 3 Sección de Estudios de Posgrado e Investigación, Escuela Superior de Medicina, Instituto Politécnico Nacional, Mexico City 11340, Mexico; jcabreraa2300@alumno.ipn.mx (J.C.C.-A.); ahernandeza2312@alumno.ipn.mx (A.L.H.-A.); 4 Department of Biological Systems, Universidad Autónoma Metropolitana-Xochimilco, Mexico City 04960, Mexico; jcastanedas@correo.xoc.uam.mx; 5 Department of Natural Sciences, Universidad Autónoma Metropolitana-Cuajimalpa, Mexico City 05348, Mexico; earechaga@cua.uam.mx; 6 Departamento de Farmacología, Instituto Nacional de Cardiología Ignacio Chávez, Mexico City 14080, Mexico; leonardo.delvalle@cardiologia.org.mx; 7 Experimental Pathology Department, Instituto Nacional de Ciencia Médicas y Nutrición “Salvador Zubirán”, Mexico City 14080, Mexico; constanzamtz@exalumno.unam.mx (C.E.M.-O.); rogelio.hernandezp@incmnsz.mx (R.H.-P.)

**Keywords:** metabolic dysfunction-associated steatotic liver disease, metabolic syndrome, dyslipidemia, lipid accumulation, oxidative stress, metformin, empagliflozin

## Abstract

Metabolic dysfunction-associated steatotic liver disease (MASLD) encompasses several cardiometabolic risk factors (obesity, insulin resistance, diabetes, and dyslipidemia), in addition to hepatic steatosis. Therefore, treatment is often challenging and frequently involves polypharmacy. This study investigated whether the combination of empagliflozin/metformin improves MASLD disease outcomes in an experimental model of metabolic syndrome (MS). To evaluate the efficacy of the empagliflozin/metformin (12.5/850 mg/kg/day/30 days) combination, male Wistar rats (200–220 g) were fed a Western-type diet and sugary drink to induce MS. Biochemical parameters, markers of liver damage, oxidative stress, and histopathological analysis were assessed. Also, the expression of transcription factors associated with carbohydrate and lipid metabolism and the modulation of oxidative stress were assessed. The analyses were performed with the combination and with the drugs independently. The combination empagliflozin/metformin decreased body weight, plasma triglycerides, and total cholesterol levels, while improving fasting blood glucose, oral glucose tolerance test, and plasma HDL-cholesterol levels. Additionally, it prevented hepatic hypertrophy, liver damage at both biochemical and histological levels, and intrahepatic lipid accumulation. The combination also demonstrated a significantly greater effect in improving mitochondrial function and reducing oxidative stress by modulating the Nrf2-mediated pathway. The empagliflozin/metformin combination therapy mitigates MASLD progression, likely by improving liver and mitochondrial function, and attenuating oxidative stress. Notably, co-therapy shows greater beneficial effects than single treatments. This protective effect appears to involve modulation of key transcription factors regulating lipid and carbohydrate metabolism, as well as influencing endogenous antioxidant defenses.

## 1. Introduction

Non-alcoholic fatty liver disease (NAFLD), the most prevalent liver disease world-wide, is characterized by hepatic triglyceride accumulation (steatosis) greater than 5%, in the absence of significant alcohol consumption [1,2]. Recently, it has been proposed to rename NAFLD to metabolic dysfunction-associated steatotic liver disease (MASLD) [3,4]. In MASLD, in addition to hepatic steatosis, at least one of the five cardiometabolic risk factors of metabolic syndrome (MS) coexist, including systemic hypertension, dyslipidemia, obesity, a proinflammatory state, hyperglycemia, insulin resistance (IR), and diabetes [3,4,5].

The clinic diagnostic of MASLD encompasses subjects with hepatic steatosis and at least one of the mentioned cardiometabolic risk factors [6,7]. A study analyzing patients’ clinical and histological features under different fatty liver disease (FLD) nomenclatures found that NAFLD describes nearly the same population as MASLD [7,8].

The worldwide NAFLD/MASLD prevalence is estimated at 29.7 to 44% in adults with significant geographic variations according to specific world regions [8,9]. The high prevalence of NAFLD/MASLD has been directly related to a global increase in high-fat and hypercaloric diet intake, as well as a sedentary lifestyle, which in turn is closely linked to an imbalance between calorie consumption and energy expenditure. This imbalance generates excess energy stored as fat in white adipose tissue, leading to obesity, high fasting plasma glucose, and IR [10]. Obesity increases the availability of plasma lipids and the uptake of free fatty acids (FFAs) within hepatocytes, promoting hepatic lipid accumulation either through β-oxidation inhibition or FFAs esterification with glycerol to form triglycerides (steatosis) [10,11,12]. Additionally, increased FFAs levels trigger inflammation by facilitating oxidative stress, likely related to mitochondrial dysfunction and increased synthesis of inflammatory cytokines and fibrosis [13].

On the other side, IR also increases de novo lipogenesis (DNL) and inhibits adipose tissue lipolysis, resulting in an enhanced FFAs influx to the liver, increased synthesis of very low-density lipoproteins (VLDLs), and reduced FFA oxidation [5,14,15]. It has been described that DNL and inhibition of FFA oxidation are related to upregulation of the transcription factor sterol regulatory element binding protein-1c (SREBP-1c) [11,15].

Regarding carbohydrate metabolism, impairment of the insulin signaling pathway decreases glycogen synthesis and induces gluconeogenesis, which contributes to high fasting plasma glucose [12,13].

Given the comorbidities associated with NAFLD/MASLD development and its worldwide prevalence, investment in prevention programs and the search for effective treatments are crucial. In this context, sustained weight loss of 5–10% and improvement in fasting glucose and insulin resistance are the most effective non-pharmacological treatments. However, available therapies primarily focus on controlling as many risk factors as possible due to the complex pathogenesis of MASLD [16,17].

The most common prescribed drugs for MASLD are antidiabetic, including insulin sensitizers, peroxisome proliferator-activated receptor (PPAR) agonists, glucagon-like peptide 1 receptor agonists (GLP-1 RA), glucagon agonists, lipogenesis inhibitors, and more recently, thyromimetics [18]. The use of antidiabetics is also supported by the fact that MASLD is regarded as an independent risk factor for, and frequently precedes the development of, type 2 diabetes mellitus (T2DM). Conversely, MASLD and diabetes often coexist in a high percentage of patients with diabetes [19].

Despite the emergence of many drugs targeting several mechanisms contributing to MASLD progression, many have failed or stalled in development because their attributed benefits are inconclusive or they induce long-term side effects. Metformin, a first-line anti-diabetic drug, improves fatty acid oxidation, inhibits lipolysis in adipose tissue and lipogenesis in the liver, and reduces hepatic gluconeogenesis, but it has limited efficacy in improving the histological features of MASLD [20,21,22]. Empagliflozin, another class of anti-diabetic drugs belonging to the sodium–glucose cotransporter type 2 inhibitor class (SGLT2i), controls blood glucose independently of insulin secretion while increasing urinary glucose excretion [23,24]. In patients with T2DM and MASLD, it also decreases body weight (2–3 kg) [24,25].

Although metformin and SGLT2i alone have shown significant benefits in diabetes, obesity, and even in cardiovascular disease, a comparative analysis in co-therapy has not been performed. It may be useful to investigate whether the combination can achieve additional benefits in preventing NAFLD/MASLD progression. Therefore, this study aims to assess the effect of empagliflozin/metformin combination therapy on MASLD progression associated with MS by evaluating its impact on risk factor control, histopathological changes, and oxidative stress.

## 2. Results

### 2.1. Validation of the Model of Metabolic Syndrome and Liver Damage

First, we validated the establishment of MS at 30 days of follow-up, before starting drug administration. We assessed body weight, lipid profile, fasting blood glucose (FBG), and OGTT in the animals fed the Western-type diet (high-fat/high-sugar diet) and sugar-sweetened beverages, and we compared them to the control. Additionally, we assessed the lipid profile by quantifying total cholesterol, triglycerides, and high-density lipoprotein cholesterol (HDL-c). All the parameters used to validate MS, as well as the markers of liver damage, were determined in the experimental groups, including the animals that were subsequently treated with the respective pharmacological treatment (untreated, combination empagliflozin/metformin, or treatments independent) (Table 1).

The group fed the Western diet and sugary drink showed increases in body weight and fasting blood glucose compared to the group fed the maintenance diet (Table 1). Additionally, the OGTT showed impaired glucose uptake (glucose intolerance). Blood glucose levels in the Western diet and sugary drink group were higher at 15, 30, and 150 min after the glucose bolus and did not return to basal levels, unlike those observed in the control group (Figure S1). The OGTT expressed as total AUC was higher in the Western diet and sugary drink group as well as in the experimental groups subsequently treated with pharmacological treatment, suggesting glucose intolerance compared with the control group fed with the maintenance diet and tap water (Table 1).

Interestingly, the western diet and sugary drink impaired the lipid profile. Increases in plasma triglycerides and total cholesterol levels were observed, while HDL-c decreased compared with the control group fed the maintenance diet (Table 1).

In short, the Western diet and sugary drink resulted in increased body weight, FBG, impairment in the lipid profile, and glucose intolerance compared with the control group fed a maintenance diet, demonstrating the successful establishment of MS. Therefore, the next aim was to assess liver function by quantifying liver enzymes as indicators of MS-induced liver dysfunction, which is closely related to the development of MASLD/NAFLD.

### 2.2. Plasma Markers of Liver Function

MS has been associated with an increased risk of MASLD development, but MASLD may also enhance several features and comorbidities of MS [14,26]. Therefore, plasma markers of liver damage were evaluated at 30 days of follow-up.

In the group fed with a Western diet and sugary drink (thereafter referred to as MASLD group), the activities of the liver enzymes alanine transaminase (ALT), aspartate aminotransferase (AST), alkaline phosphatase (ALP), γ-Glutamyl transferase (GGT), and bilirubin total (TB) and direct (DB) in plasma were increased compared with the group fed the maintenance diet (Table 1). Also, in the groups fed Western diet and sugary drink which were assigned to the pharmacological treatments, the liver damage markers were increased in comparison to the control group, with no significant differences between them (Table 1).

The results over 30 days demonstrate an increase in body weight (indicative of obesity) and fasting blood glucose, as well as glucose intolerance and an impairment in the lipid profile, which are risk factors for both MS and MASLD. Additionally, the plasma markers of liver function were increased, suggesting liver injury and likely MASLD. Therefore, the next aim was to assess whether the empagliflozin/metformin combination was effective in preventing or delaying the progression of fatty liver disease.

### 2.3. Effect of Empagliflozin/Metformin Combination on Risk Factors Associated with MASLD Progression

The therapeutic effect of the combination on MASLD progression was first assessed by examining its impact on risk factors.

**Figure 1:**
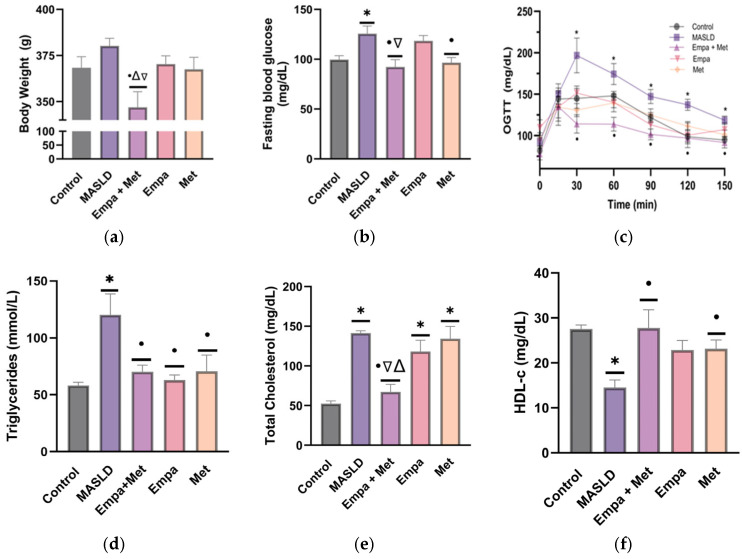
Effect of Empa + Met combination on risk factors associated with MASLD: (**a**) body weight; (**b**) fasting blood glucose; (**c**) oral glucose tolerance test [area under curve (AUC)]; (**d**) plasma triglycerides; (**e**) plasma total cholesterol; and (**f**) plasma HDL-c. Empagliflozin (Empa), metformin (Met), empagliflozin + metformin (Empa + Met); metabolic dysfunction-associated steatotic liver disease (MASLD). Data are expressed as mean ± SEM, *n* = 6, and were analyzed by one-way ANOVA. Statistical significance was established as * *p* < 0.05 vs. control; ^•^ *p* < 0.05 vs. MASLD, ^▽^ *p* < 0.05 vs. Empa; ^Δ^ *p* < 0.05 vs. Met.

At the end of the study, the MASLD group (induced by the diet) showed an increase in FBG compared with the control group. The group that received the combined treatment showed a decrease in body weight and fasting blood glucose compared with the MASLD group (Figure 1a,b).

Administration of empagliflozin and metformin alone did not prevent body weight gain compared with the combined treatment group, and only empagliflozin lowered FBG compared with the MASLD group (Figure 1a,b). The MASLD group also showed impaired glucose uptake, as demonstrated by the OGTT, which was impaired and in the MASLD group, the blood glucose did not decrease to the basal levels (Figure 1c). Area under curve was higher in the MASLD groups than in the control group (Figure S2). This impairment was significantly decreased with the empagliflozin/metformin combination compared with the single-agent treatments (Figure 1c), suggesting glucose intolerance.

Regarding the effects of the empagliflozin/metformin combination on the lipid profile, the MASLD group showed increased plasma triglycerides and total cholesterol, while HDL-c was decreased compared with the control group. The treatments were effective in decreasing TG compared with the MASLD group, but only the combination was effective in decreasing total cholesterol (Figure 1d,e). In addition, plasma HDL-c levels were also effectively restored with the combination therapy compared with the MASLD group (Figure 1f).

### 2.4. Effect of the Combination Empagliflozin/Metformin on the Markers of Liver Damage in MASLD

**Figure 2:**
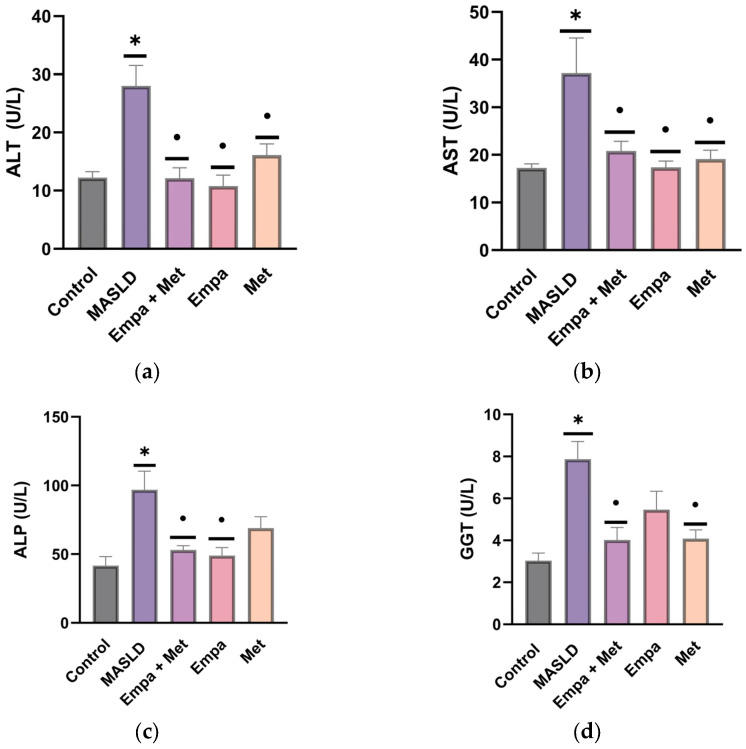

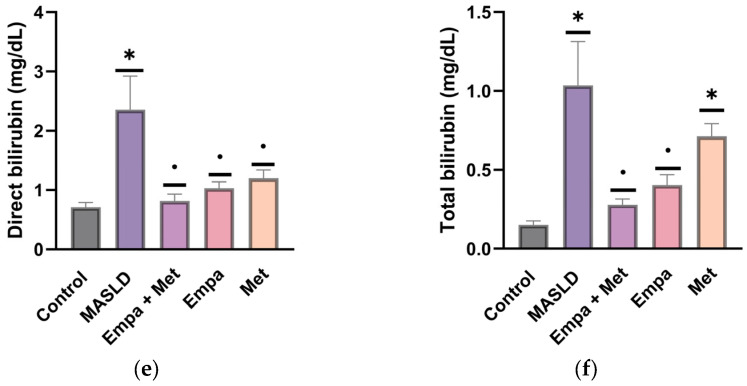
Effect of the treatments on the liver damage markers in plasma of the MASLD experimental model: (**a**) alanine transaminase (ALT); (**b**) aspartate aminotransferase (AST); (**c**) alkaline phosphatase (ALP); (**d**) γ-Glutamyl transferase (GGT); (**e**) direct bilirubin; (**f**) total bilirubin. empagliflozin (Empa), metformin (Met), empagliflozin + metformin (Empa + Met); metabolic dysfunction-associated steatotic liver disease (MASLD). Data are expressed as mean ± SEM, *n* = 6, analyzed by one-way ANOVA. Statistical significance was established as * *p* < 0.05 vs. control; ^•^ *p* < 0.05 vs. MASLD.

At 60 days of follow-up, liver damage markers remained significantly higher in the MASLD group compared with the control group (Figure 2a–f), showing a similar trend to that observed at 30 days. All treatments significantly reduced the levels of liver markers (ALT, AST, ALP, GGT, TB, and DB), but combination therapy was the most effective, particularly in the reduction of ALP and GGT, when compared with the monotherapy (Empa or Met) groups and the MASLD group (Figure 2a–d). Empagliflozin and metformin alone showed a low protective effect compared to the combination in GGT, ALP, and TB, respectively.

### 2.5. Liver Hypertrophy

**Figure 3:**
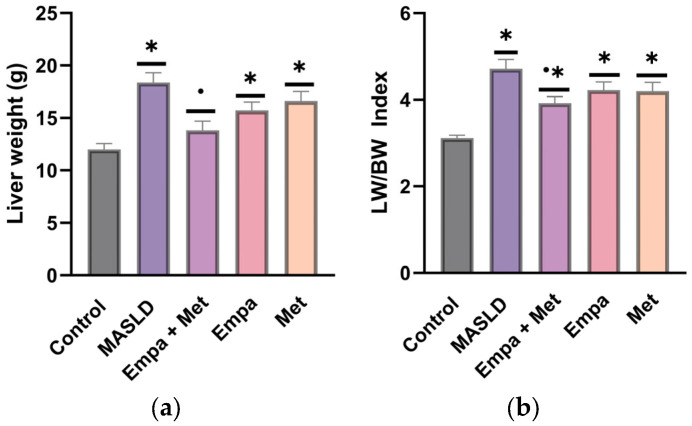
Effect of the treatments on liver weight: (**a**) liver weight; (**b**) liver weight/body weight index (Ratio LW/BW). Empagliflozin (Empa), metformin (Met), empagliflozin + metformin (Empa + Met); metabolic dysfunction-associated steatotic liver disease (MASLD). Data are expressed as mean ± SEM, *n* = 6, analyzed by one-way ANOVA. Statistical significance was established as * *p* < 0.05 vs. control; ^•^ *p* < 0.05 vs. MASLD.

Although liver hypertrophy is not considered a specific marker of liver damage, it can be a sign of injury that leads to an initial hepatic response to inflammation and tissue destruction. After sacrifice, we determined liver weight and calculated the liver weight/body weight ratio as an index of hypertrophy. We observed that animals with MASLD presented significant increases in both liver weight and the LW/BW ratio (Figure 3). While all treatments reduced liver weight and the hypertrophy index, the empagliflozin/metformin combination was the most effective when compared to MASLD group (*p* < 0.05) (Figure 3).

### 2.6. The Combination Empa + Met Alleviates Liver Damage and Hepatic Lipid Accumulation in Metabolic Dysfunction-Associated Steatotic Liver Disease (MASLD)

To assess the effect of the empagliflozin/metformin combination on liver damage and lipid accumulation in hepatocytes, we analyzed liver slides stained with H&E and oil red O.

**Figure 4:**
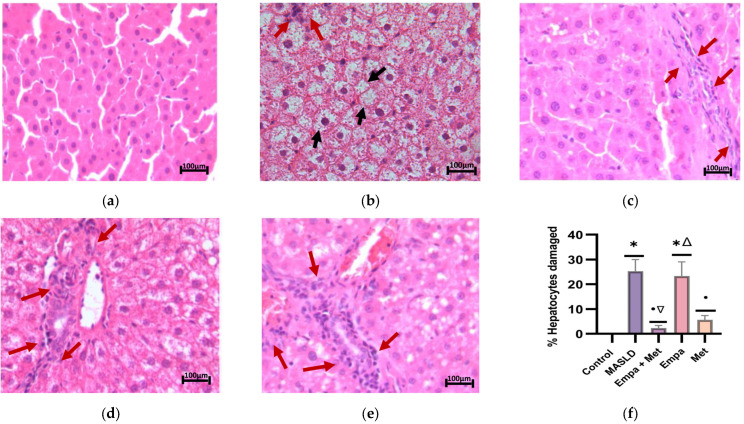
Histopathological analysis of the combination empagliflozin + metformin: (**a**) control; (**b**) MASLD; (**c**) Empa + Met; (**d**) Empa; (**e**) Met; (**f**) % Hepatocytes damaged. Black arrows indicate hepatocyte damaged and red arrows indicate inflammatory infiltrate. Empagliflozin (Empa), metformin (Met), empagliflozin + metformin (Empa + Met), metabolic dysfunction-associated steatotic liver disease (MASLD). All sections hematoxylin/eosin staining, 200× magnification. All sections hematoxylin/eosin staining, 200× magnification. Data are expressed as mean ± SEM, *n* = 3, analyzed by one-way ANOVA. Statistical significance was established as * *p* < 0.05 vs. control; ^•^ *p* < 0.05 vs. MASLD, ^▽^ *p* < 0.05 vs. Empa; ^Δ^ *p* < 0.05 vs. Met.

H&E staining showed that liver tissue of the control group had a normal structure with well-ordered hepatocyte cords and well-defined liver lobules, with no hepatic steatosis observed (Figure 4a). In contrast, the micrograph of the MASLD group showed macrovesicular steatosis as a characteristic pattern on H&E staining, where hepatocytes contained a single large vacuole displacing the nucleus towards the cell periphery. The empty spaces in the hepatocytes were due to the presence of fat dissolved during preparation (Figure 4b). In the group treated with the empagliflozin/metformin combination (Figure 4c), significantly reduced hepatocyte steatosis was observed, with a reduction or elimination of lipid droplets and cytoplasmic vacuolization. This reduction was less significant in the Empa and Met groups, although some improvement was observed (Figure 4d,e). Figure 4f shows the comparative quantitative analysis of damaged hepatocytes. Although a decrease in the percentage of damaged hepatocytes was observed with the different treatments, Empa + Met combination showed a significant reduction in liver damage compared with the MASLD group (Figure 4f).

The presence of NASH was determined using the NAFLD activity scoring system (NAS), which is based on the sum of three key histological components such as hepatocytes in ballooning degeneration, apoptosis, and lobular inflammation.

**Figure 5:**
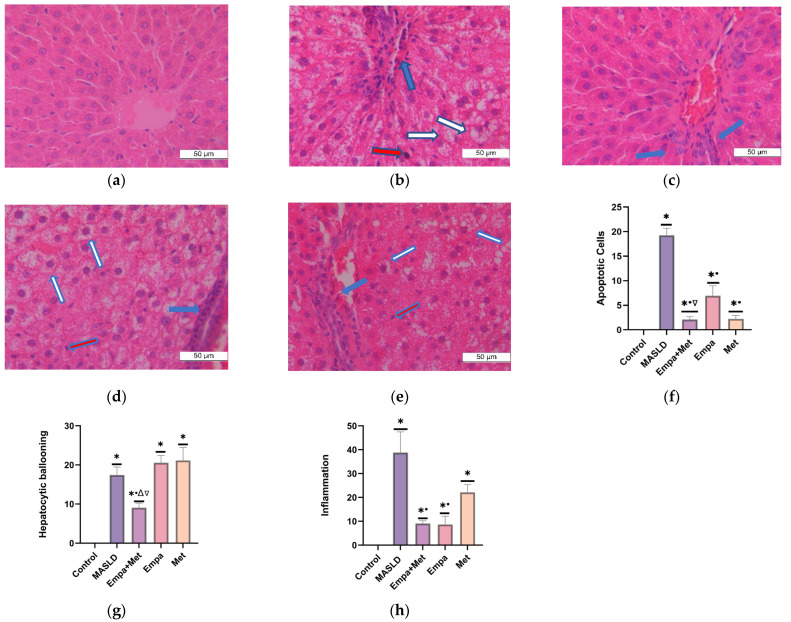
Histopathological analysis of the combination empagliflozin + metformin: (**a**) control; (**b**) MASLD; (**c**) Empa + Met; (**d**) Empa; and (**e**) Met. Quantitative analysis of apoptotic cells (**f**); hepatocytic ballooning (**g**), and inflammation (**h**). Red arrows indicate hepatic apoptosis, blue arrows indicate presence of inflammatory cell infiltrate and white arrows indicate balloonised hepatocytes. Empagliflozin (Empa), metformin (Met), empagliflozin + metformin (Empa + Met), metabolic-associated steatotic liver disease (MASLD). All sections hematoxylin/eosin staining, 400× magnification. Data are expressed as mean ± SEM, *n* = 3, analyzed by one-way ANOVA. Statistical significance was established as * *p* < 0.05 vs. control; ^•^ *p* < 0.05 vs. MASLD, ^▽^ *p* < 0.05 vs. Empa; ^Δ^ *p* < 0.05 vs. Met.

The MASLD group showed an increased presence of hepatic apoptosis, characterized by hepatocytes with condensed nuclei showing a darker coloration (red arrow). In addition, markedly larger hepatocytes with lighter and less dense cytoplasm (hepatocyte ballooning) (white arrow) and the exacerbated presence of mixed inflammatory cells in the hepatic lobule (blue arrow) were observed (Figure 5b). A significant increase (*p* < 0.05) in the three components in the MASLD group when compared to individual treatments and co-therapy, which demonstrated that the Empa + Met combination was the most effective therapy, with the most significant reduction in the number of apoptotic cell/fields, fewer ballooning hepatocytes and less lobular inflammation (Figure 5f–h).

The degree of NASH activity and presence of fibrosis in the MASLD model were determined according to the score reported by Brunt E.M., and Tiniakos D.G., 2010 [27]. Control Group: No NASH; MAFLD Group: Severe NASH; Empa + Met Group: Moderate NASH; Empa Group: Moderate NASH; Met Group: Moderate NASH.

**Figure 6:**
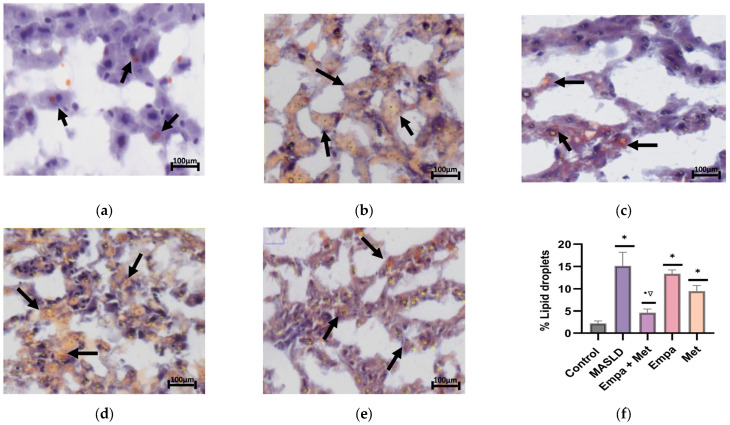
Analysis of the effect of the empagliflozin + metformin combination on the lipids accumulation in the liver: (**a**) control; (**b**) MASLD; (**c**) Empa + Met; (**d**) Empa; (**e**) Met; (**f**) quantitative analysis of lipid droplets. Empagliflozin (Empa), metformin (Met), empagliflozin + metformin (Empa + Met), metabolic dysfunction-associated steatotic liver disease (MASLD). Black arrows indicate lipid droplets. All sections oil red staining, 50× magnification. Data are expressed as mean ± SEM, *n* = 3, analyzed by one-way ANOVA. Statistical significance was established as * *p* < 0.05 vs. control; ^•^ *p* < 0.05 vs. MASLD, ^▽^ *p* < 0.05 vs. Empa.

Oil-red O staining was performed to demonstrate lipid droplets within the hepatocytes, and the results are expressed as a percentage per field. Figure 6a depicts the stain of a representative sample from the control group, where a few faint orange-positive stains indicate minimal lipid deposition. As can be observed in the MASLD group, a higher intensity of orange-positive stains indicates greater lipid deposition than in the control group (Figure 6b). The combination treatment reduced the positive staining, demonstrating a decrease in lipid accumulation (Figure 6c). The single-agent treatments also reduced lipid accumulation but were not as effective as the combination (Figure 6f). A summary of this assay can be clearly observed in Figure 6f. These results are consistent with the plasma parameters, where the Empa + Met treatment showed significant improvements in total cholesterol, triglycerides, and HDL-c.

Quantitative analysis of hepatic triglycerides and cholesterol content in the experimental MASLD group, as well as the groups treated with the empagliflozin/metformin combination and the treatments independently, demonstrated an increase in the triglycerides and cholesterol content in the MASLD group when compared with the control group (Figure S3a,b). The empagliflozin/metformin combination successfully reduced the content of triglycerides in the liver when compared with the untreated MASLD group, but the levels were still higher than in the control group. Of the single agent treatments, only metformin showed a reductive effect of triglycerides content. Regarding the effects of the treatments on cholesterol accumulation, a trend toward decreased cholesterol content was observed in the groups treated with the combination and metformin, but without significant differences when compared with the untreated group (Figure S3).

### 2.7. Effect of the Empagliflozin/Metformin Combination on Oxidative Stress in Liver Tissue

As previously described, the development and progression of MASLD to advanced stages involve oxidative stress. Therefore, the effect of the Empa + Met combination was assessed on oxidized proteins and lipids by quantifying 2,4-dinitrophenylhydrazine (DNPH) and malondialdehyde (MDA) as indirect measurement of oxidative stress in the liver.

**Figure 7:**
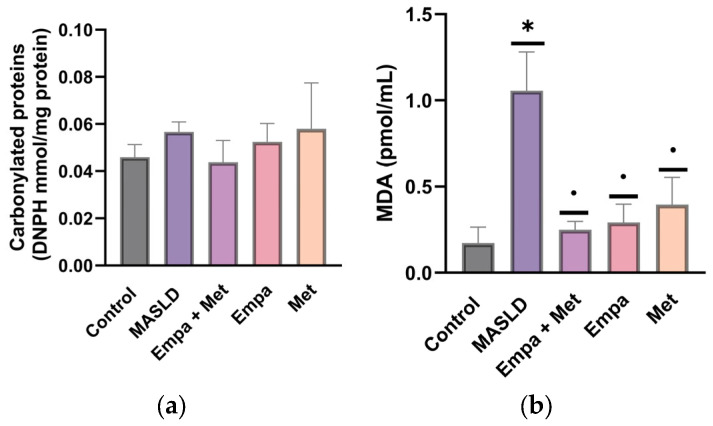
Effect of the combination empagliflozin/metformin on oxidative stress in the liver: (**a**) oxidized proteins; (**b**) oxidized lipids. DNPH: 2,4-dinitrophenylhydrazine; MDA: malondialdehyde; empagliflozin (Empa), metformin (Met), empagliflozin + metformin (Empa + Met), metabolic dysfunction-associated steatotic liver disease (MASLD). Data are expressed as mean ± SEM, *n* = 6, analyzed by one-way ANOVA. Statistical significance was established as * *p* < 0.05 vs. control; ^•^ *p* < 0.05 vs. MASLD.

Oxidized proteins in the MASLD group tended to be increased compared with the control group, and the empagliflozin/metformin combination showed a trend toward a decrease, though neither case reached statistical significance (Figure 7a). Notably, oxidized lipids were increased in the livers of MASLD group, and all treatments were effective in reducing lipid oxidation compared with the untreated group (Figure 7b).

### 2.8. Effect of Empagliflozin/Metformin Combination on Mitochondrial Complexes I and II Activity

The main function of mitochondria is ATP synthesis through the oxidation of various substrates and through the electron transport chain, which creates an electrochemical gradient by the electron flow in the mitochondrial complexes. However, disruptions to the flow of electrons in the early stages of the electron transfer chain can result in an increase in ROS, free radical production, and oxidative stress. Therefore, to explain the observed increase in lipid oxidation, we assessed the function of mitochondrial complexes I and II.

**Figure 8:**
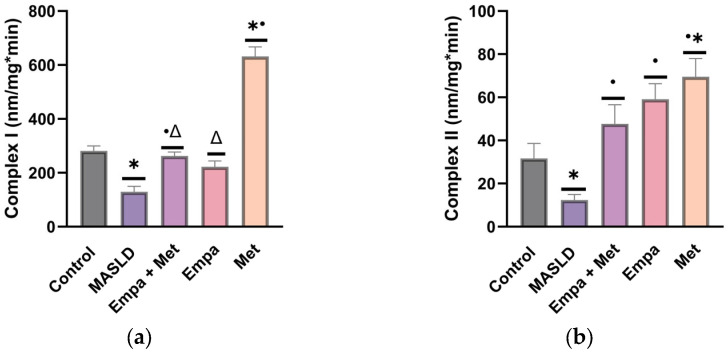
Mitochondrial complexes activities: (**a**) Complex I; (**b**) Complex II. Empagliflozin (Empa), metformin (Met), empagliflozin + metformin (Empa + Met), metabolic dysfunction-associated steatotic liver disease (MASLD). Data are expressed as mean ± SEM, *n* = 6, analyzed by one-way ANOVA. Statistical significance was established as * *p* < 0.05 vs. control; ^•^ *p* < 0.05 vs. MASLD, ^Δ^ *p* < 0.05 vs. Met.

The MASLD group showed a significant decrease (*p* < 0.05) in the activity of both mitochondrial complexes (I and II) compared with the control group (Figure 8). Compared with the MASLD group, we observed improved activity of both mitochondrial complexes, in the co-therapy group as well as in the single-therapy groups (Figure 8). These results support the hypothesis that, in MASLD progression, mitochondrial dysfunction promotes ROS production through the uncoupling of the mitochondrial complexes.

### 2.9. Effect of the Combination Empagliflozin/Metformin on the Endogenous Antioxidant Enzymes in the Liver

Maintaining redox balance is important to prevent the increased oxidative stress and involves several antioxidant systems, including endogenous ones such as glutathione reductase (GR), glutathione peroxidase (GPX), and superoxide dismutase (SOD).

**Figure 9:**
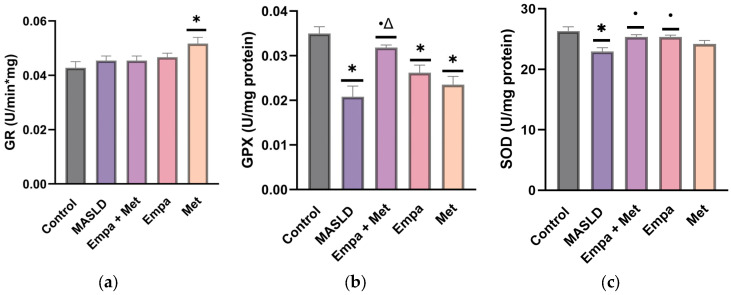
Activities of the endogenous antioxidant enzymes in the liver: (**a**) glutathione reductase (GR), (**b**) glutathione peroxidase (GPX), and (**c**) superoxide dismutase (SOD). Empagliflozin (Empa), metformin (Met), empagliflozin + metformin (Empa + Met), metabolic dysfunction-associated steatotic liver disease (MASLD). Data are expressed as mean ± SEM, *n* = 6, analyzed by one-way ANOVA. Statistical significance was established as * *p* < 0.05 vs. control; ^•^ *p* < 0.05 vs. MASLD; ^Δ^ *p* < 0.05 vs. Met.

As Figure 9a depicts, GR activity did not differ significantly between any of the groups evaluated. However, the activities of GPX and SOD were impaired in the livers of the MASLD group (Figure 9b,c). We observed that all the treatments, especially Empa + Met combination, improved GPX and SOD activity (Figure 9b,c), indicating that co-therapy helped restore levels of these antioxidant enzymes compared with the MASLD group (Figure 9b,c).

### 2.10. Expression of Transcription Factors Involved in Lipid Metabolism and Oxidative Stress in the Liver

The empagliflozin/metformin combination, as well as the individual treatments effectively improved biochemical parameters, liver damage markers, intrahepatic lipid content, mitochondrial complex function, and oxidative stress. To explore the cellular mechanisms involved in these benefits, the nuclear translocation of sterol regulatory element-binding protein (SREBP), carbohydrate responsive element-binding protein (ChREBP), and nuclear factor-erythroid 2 p45-related factor 2 (Nrf2) were assessed in nuclear extracts of liver homogenates.

**Figure 10:**
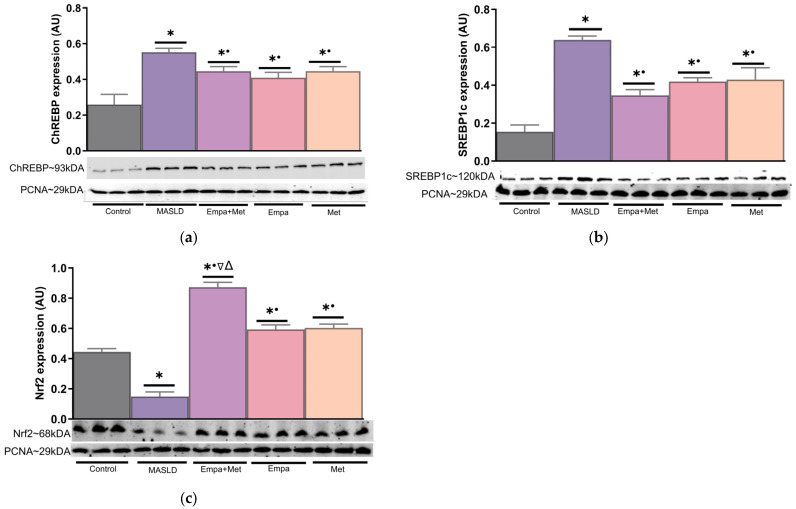
Expression of transcription factors associated with the modulation in the expression of proteins involved in lipid and carbohydrate metabolism and endogenous antioxidant system. (**a**) Liver nuclear expression of ChREBP; (**b**) liver nuclear expression of SREBP1c; and (**c**) liver nuclear expression of Nrf2. Empagliflozin (Empa), metformin (Met), empagliflozin + metformin (Empa + Met), metabolic dysfunction-associated steatotic liver disease (MASLD). For representative Western blotting, 3 randomly selected samples per group were analyzed, respectively. Data are expressed as mean ± SEM, analyzed by one-way ANOVA. Statistical significance was established as * *p* < 0.05 vs. control; ^•^ *p* < 0.05 vs. MASLD, ^▽^ *p* < 0.05 vs. Empa; ^Δ^ *p* < 0.05 vs. Met.

The nuclear translocation of lipogenesis-related transcription factors ChREBP and SREBP1c was increased in the livers of the MASLD group compared with the control group (Figure 10a,b). The empagliflozin/metformin combination reduced the expression of both transcription factors compared with the MASLD group, although the reduction was not as pronounced as in the control group. The individual treatments also prevented the increase in ChREBP and SREBP1c transcription factors nuclear translocation (Figure 10a,b). In contrast, Nrf2 transcription factor expression in the livers of MASLD group was decreased compared with the control group (Figure 10c). The empagliflozin/metformin combination prevented this decrease in Nrf2 expression; its expression was even higher than the control group. The individual treatments also restored Nrf2 expression in the liver (Figure 10c).

## 3. Discussion

The aim of this study was to determine the effect of a combination of drugs used in T2DM therapy (Empa + Met) on the progression of MASLD/NAFLD. To address this question, we used an experimental model of MS induced with a Western-type diet (high-fat/high-sugar diet) and sweetened beverage (3.8% glucose, 7.2% fructose), which induced several well-known risk factors that favor the development of MASLD [28,29,30,31]. Ad libitum feeding of a high-fat diet and consumption of corn syrup-based (fructose/glucose) sugar-sweetened beverages have been reported to increase intrahepatic free fatty acids accumulation, visceral adipose tissue, lipogenesis, and impaired fat oxidation. In addition, this regimen exacerbates MASLD through lipotoxicity, increased oxidative stress, and the release of inflammatory cytokines [32,33,34]. In our study, metabolic alterations were established after 30 days of a Western-type diet and the ingestion of a sweetened beverage, allowing us to initiate intervention studies. One limitation of our study is the absence of an experimental group that underwent a 30-day period to confirm the presence of lipid accumulation in hepatocytes. We hypothesized that MASLD was established based on the presence of metabolic risk factors and the elevated markers of liver damage which align with clinical criteria for diagnosing this condition [3,4,5,6,7].

Thus, we studied the empagliflozin/metformin combination because both drugs are well-known, and we hypothesized that the combination would be useful in MASLD treatment, where a plethora of cardiometabolic risk factors coexist, including glucose intolerance, dyslipidemia, obesity, and IR. Moreover, both drugs are commercially available and easily accessible, and their short- and long-term side effects are reasonably well-known and characterized.

In this study, the empagliflozin/metformin combination improved FBG, OGTT, TC, TG, HDL-c, and decreased ALT, AST, GGT, ALP, TB, and DB. In the MASLD group, we observed steatosis and inflammatory infiltrates, while the group treated with Empa + Met showed a clear improvement in these markers. Histologically, there was a reduction in inflammatory cells and a lower percentage of lipid droplets in the liver tissue. Other studies have reported that the empagliflozin/metformin combination reduced ALT levels, body weight, and glycosylated hemoglobin, and significantly improved fibrosis and ultrasound steatosis grades compared to metformin alone [35,36,37]. Also, the use of SGLT2i alone has been shown to reduce hepatic lipid accumulation and improve serum liver damage markers [3,38]. However, other studies in patients with T2DM treated with combined empagliflozin (10 and 25 mg) and metformin have shown no significant differences in the beneficial effects (Hb1Ac, body weight, systolic blood pressure, diastolic blood pressure, or lipid profile) or side effects between the two empagliflozin doses [39].

Concurrent with MASLD progression, oxidative stress occurs due to ROS production and antioxidant defense dysregulation. We observed decreased activity of mitochondrial complex I and II in the MASLD group, indicating a reduced oxidative capacity, possibly increasing electron leakage, and increased ROS production (superoxide or hydrogen peroxide) [40]. The complexes I and II facilitate the transfer of electrons from NADH and FADH, respectively, to generate an electrochemical gradient for ATP synthesis. Dysfunction in these complexes disrupts the electron flow in the early stages of the electron transfer chain, resulting in an increase in ROS and free radical production. Moreover, in MASLD, lipid accumulation drives ROS production and oxidative stress, impairing mitochondrial function and exacerbating liver damage by activating inflammatory pathways and apoptosis [41]. This hepatic lipid accumulation can progress to metabolic dysfunction-associated steatohepatitis (MASH), a more severe condition that can ultimately lead to cirrhosis and hepatocellular carcinoma through the interplay of inflammation, oxidative stress, and fibrosis [23,42,43]. In this context, our data showed higher ballooning, apoptosis, and inflammation in the liver of the MASLD group, which were decreased with the empagliflozin/metformin combination. However, in the group treated with metformin alone, the inflammation was higher than the other groups. Although inflammation is often associated with liver damage, portal inflammation has been suggested as evidence of histological resolution of NASH independent of the type of therapeutic intervention (medical, dietary, or surgical) [44]. This feature has been confirmed in clinical trials. In patients with T2DM, Resmetirom (Rezdifra, the first FDA-approved treatment for NASH) at two doses (80 and 100 mg/day/52 weeks) showed resolution of NASH and improvement in fibrosis, but portal inflammation persisted or increased [45]. Another phase III clinical study referred to as ‘Stellar’ evaluated Selonsertib (SK1 inhibitor) in patients with fibrosis or cirrhosis compensated, the results showed no improvement in liver fibrosis without worsening of NASH, despite pharmacological treatment, suggesting that persistent inflammation, which was not controlled by the treatment, is part of the pathological mechanism of the disease, even is necessary for tissue healing and repair, and often is present during the early stages of liver injury [46,47].

Furthermore, oxidative stress in MASLD triggers lipid peroxidation of specific polyunsaturated fatty acids (PUFAs), along with the formation of highly reactive aldehyde products such as MDA. In our study, we observed increase in MDA in the liver, but the activity of GPx and SOD were decreased in the MASLD group. These enzyme activities were restored with empagliflozin/metformin combination. Other studies reported that in patients with T1DM and T2DM, the combination of an SGLT2i and metformin increased levels of the antioxidants (TAS, SOD, and GPx), and prooxidants [AOPP, isoprostanes, and advanced glycation end-products (AGEs)], as well as inflammatory parameters [C-reactive protein (CRP), IL-6], but also reduced NAFLD fibrosis score [48,49,50]. These studies also observed improvement in liver damage markers and fat content. Notably, urogenital infections, diabetic ketoacidosis, or hypoglycemia (mild or severe) were observed during those studies [48,49,50].

In our study, the combination therapy reduced oxidative stress and increased the activity of endogenous antioxidant enzymes. Such antioxidant effect was mediated by increasing Nrf2 nuclear translocation the key master transcription factor associated with the endogenous antioxidant response [51]. Other studies have reported that SGLT2i increases renal Nrf2 nuclear translocation in the kidney of diabetic animals [52]. In contrast, studies in experimental models of steatosis have reported decreased in Nrf2 expression, whereas increased hepatic antioxidant activity is associated with Nrf2; conversely loss of Nrf2 activity exacerbates steatohepatitis [53,54]. To our knowledge, no studies in the liver have yet demonstrated that the combination therapy stimulates Nrf2 translocation and expression which is a key responsible antioxidant mechanism.

Furthermore, SGLT2i improved hepatic steatosis by decreasing NLRP3 (nucleotide-binding domain, leucine-rich repeat-containing family, pyrin domain containing 3) inflammasome activation, significantly inhibiting the production of interleukin (IL)-1, IL-6 or tumor necrosis factor-alpha (TNF-alpha), and improving hepatic steatosis [55,56]. Another beneficial mechanism of SGLT2i involved in regulating lipid and carbohydrate metabolic pathways is the activation of adenosine 5-monophosphate (AMP)-activated protein kinase alpha (AMPK), and inhibition of 6-phosphofructo-2-kinase (PFK2), which helps attenuate the activation of inflammatory processes by inhibiting glucose metabolism [41,56].

On the other hand, modulation of carbohydrates and lipid metabolism plays a key role in pathogenesis and progression of steatosis. In DNL, insulin and glucose activate SREBP-1c and ChREBP, both transcription factors responsible for regulating FAS, ACC, and stearoyl-CoA desaturase 1 (SCD1), which are involved in lipogenesis [15]. Thus, we assessed the nuclear expression of ChREBP and SREBP1c and observed an increase in both transcription factors in MASLD, which were downregulated with empagliflozin/metformin combination. Consistent with our data, in experimental MASLD, dapagliflozin reduced DNL by upregulating nuclear receptors farnesoid X receptor/small heterodimer partner (FXR/SHP) and downregulating liver X receptor alpha (LXRα)/SREBP-1c in the liver. Other studies reported that dapagliflozin reduced inflammation by inhibiting the nuclear factor kappa-light-chain-enhancer of activated B cells (NF-κB) pathway and alleviated fibrosis by restoring the balance between fibrogenesis and fibrolysis in the liver [57].

We observed greater beneficial effects with the combined therapy than with metformin alone. Metformin, however, has been reported to improve lipid profiles and insulin metabolism, reduce body weight, and decrease biochemical markers of liver disease in patients with NAFLD [22,58,59]. The hepatoprotective effect of this drug has been attributed to the specific activation of AMPK and subsequent suppression of DNL through increased phosphorylation of acetyl-CoA carboxylase 1 (ACC1), an enzyme involved in the conversion of acetyl-CoA to malonyl-CoA [60,61]. It has also been suggested that metformin suppresses the transcription of Srebp-1c by inhibiting endogenous liver X receptor (LXR) [62,63]. Regarding anti-inflammatory effects, metformin inhibits TNF-α production through the AMPK-sirtuin 1 (Sirt1) signaling pathway in hepatocytes and Kupffer cells (KCs) [61].

Notably, the empagliflozin/metformin combination has been reported to inactivate p38 MAPKα and ERK1/2 activity, with empagliflozin specifically enhancing AMPK-induced NF-κB inactivation (the mechanism of action of metformin), thus preventing liver inflammation and fibrosis [64]. Furthermore, an in silico study suggests that the combination may interact with TLR2 and DECTIN1 receptors, and increase the expression of *Tlr2* and *Clec7* to directly modulate inflammatory gene expression in macrophages and upregulate the expression of their receptors during diabetes [65].

Despite the emergence of many drugs directly targeting several mechanisms contributing to MASLD progression, some have failed or stalled in development due to inconclusive benefits or long-term side effects. Treating a disease characterized by multiple pathogenic mechanisms poses significant challenges difficult, and often involves the use of polypharmacy. Consequently, there is a critical need to explore drug combinations that can effectively manage a range of risk factors while simultaneously regulating glucose and lipid metabolism. This approach could play an important role in preventing or delaying inflammation, oxidative stress, and the advance of liver fibrosis [66].

To the best of our knowledge, our study is the first to investigate the combined therapy of the antidiabetics empagliflozin/metformin in MASLD. Furthermore, we aimed to extrapolate our experimental model to the clinical context. Thus, we first established metabolic alterations and assessed liver damage markers before initiating treatments, mimicking the clinical progression in patients. Moreover, we allowed ad libitum food and drink intake, reflecting the often-limited adherence to restrictive diets in patients. Based on our results, the empagliflozin/metformin combination could be a highly effective option for controlling risk factors and preventing MASLD progression by modulating oxidative stress and inflammation and possibly improving mitochondrial function. Further research should explore the systemic benefits of this combination, including its effects on other target organs affected by these risk factors, such as the kidney, heart, and vascular system.

Based on the results obtained, we hypothesize that the beneficial effects were mainly conferred by empagliflozin, since SGLT2i are used to improve blood glucose control and prevent the risk of cardiac and renal complications in T2DM patients. They are commonly prescribed as an adjunct to lifestyle changes, including diet and exercise. Some benefits of SGLT2i use include their non-insulin-dependent antidiabetic effects, resulting in a low risk of hypoglycemia. Nevertheless, hypoglycemia can occur when SGLT2i is prescribed in combination with insulin or any insulin-dependent or sensitizing antidiabetic medication [67]. Due to glucose excretion in the urine, gluconeogenesis is activated, increasing glucagon levels and reducing insulin levels, as anabolic metabolic pathways are modulated by insulin. Lipolysis and ketogenesis are also induced, increasing substrates that improve mitochondrial function and reduce oxidative stress [67].

The successful therapeutic strategy in our study is attributed to the combination therapy which appeared safe and well tolerated. The animals were not hyperglycemic or diabetic, and we did not detect hypoglycemia.

Although we utilized a relatively high dose, it was found to be safe and well tolerated, with no hypoglycemia detected. It is noteworthy that combination therapy controls more risk factors in MASLD than individual treatments. Despite its positive findings, our study has some limitations. We did not include a 30-day experimental group to obtain the liver tissue and directly demonstrate the presence of lipid accumulation in hepatocytes and assess the progression in the MASLD group. We also did not include a placebo group to determine the treatments’ true efficacy nor analyze drug interactions. We used empagliflozin/metformin at a dose of 12.5/850 mg/kg/day, a dose prescribed for patients with diabetes. We did not assess lower or higher doses, so we do not know whether other doses might be more effective. Another limitation is that we did not analyze the expression of FAS, ACC, and SCD1, responsible for DNL. Therefore, future studies could explore the signaling pathways involved in lipid and carbohydrate metabolism. We used a single dose; therefore, we did not assess morning/night dosing, which should also be considered to maximize health benefits. Therefore, we do not know whether twice-daily treatment might be more effective. The inflammatory cell types in the liver were not identified, which would be useful in determining whether the treatments polarize any specific type of inflammatory cell. Also, future research could analyze statistical interactions to clarify any type of interaction between empagliflozin and metformin, if present. Finally, other drug combinations should be assessed to demonstrate the efficacy of empagliflozin/metformin further.

Despite these limitations, we conclude that empagliflozin/metformin combination showed a significantly greater effect in delaying MASLD progression than either monotherapy. However, further studies are needed to elucidate the cellular mechanisms underlying its benefits and better understand potential long-term side effects. Our findings set standards for future studies using this combination in patients without diabetes but with metabolic risk factors, where exploratory investigations of lower doses, potential adverse effects, and the cellular mechanisms underlying its benefits can be conducted.

## 4. Materials and Methods

### 4.1. Ethics Statement

All experiments were performed using male Wistar rats in accordance with the Mexican Federal Regulation for Animal Care and Experimentation (NOM-062-ZOO 2001) and were approved by the Institutional Animal Care and Use Committee (CICUAL) and Investigation Committee of Instituto Nacional de Cardiología Ignacio Chávez (approved by CICUAL with the Registration Number INC/CICUAL/002/2022 and Institutional Registration number 21-1290, respectively).

### 4.2. Experimental Design

Thirty male Wistar rats (200–220 g, 4 weeks old) were used. The vivarium of the Instituto Nacional de Cardiología Ignacio Chávez provided these rats. All rats were kept under controlled conditions with 12 h artificial light/dark cycles and a temperature of 22 ± 3 °C. Diet and water were provided ad libitum according to their respective group/treatment. The rats were randomly divided into 2 groups at the beginning of the study: control group (Control) (*n* = 6) receiving a maintenance diet (Labdiet 5001 Rodent Diet^TM^) and tap water, and the metabolic syndrome group (MS) (*n* = 24) receiving a Western-type diet (Table S1) and sweetened beverage (3.8% glucose, 7.2% fructose) [28,30]. Both experimental groups were maintained for 30 days with ad libitum access to the beverage and food.

MS was considered successfully established when at least three of the following parameters increased by 20% compared to the control group: body weight, systolic blood pressure (SBP), fasting blood glucose (FBG), glucose intolerance, and dyslipidemia [triglycerides (TGs), total cholesterol (TC), and high-density lipoprotein-cholesterol (HDL-c)]. Animals that did not meet these criteria were excluded from the study. Following validation of metabolic syndrome, the animals were allocated homogeneously across all experimental groups to minimize bias before initiating the treatments.

After MS diagnosis (30 days) and after demonstrate liver dysfunction through the determination of liver damage markers, the MS group was further divided into four subgroups: an untreated MASLD group, a MASLD group treated with empagliflozin + metformin [12.5/850 mg/Kg body weight (B.w.)/day] (Empa + Met), a MASLD group treated with empagliflozin (12.5 mg/Kg B.w./day) (Empa), and a MASLD group treated with Metformin (850 mg/Kg B.w/day) (Met). The treatments were resuspended in 1% hydroxyethyl cellulose (HEC) and administered daily by gastric gavage for 30 days in the morning. The untreated MASLD and control groups received a similar volume of the vehicle (HEC).

After completing the experimental protocols (a total of two months), the animals were anesthetized with sodium pentobarbital (65 mg/kg i.p.) and exsanguinated by abdominal aorta puncture. Whole blood was deposited in tubes containing heparin as an anticoagulant to prevent clotting, followed by centrifugation at 2000× *g* for 10 min in a refrigerated centrifuge to separate the cellular components from the liquid plasma layer. Plasma samples were collected and frozen at −70 °C until further analysis.

The livers were then surgically removed and placed in cold NaCl 0.9% to remove blood debris. All tissue samples were snap-frozen in liquid nitrogen and stored at −70 °C until further analysis.

### 4.3. Blood Samples

After an overnight fast at two time points (30 and 60 days), a blood sample was obtained from the tail vein. 0.5 milliliters of blood were drawn after aseptic preparation and collected into heparinized tubes to obtain plasma. Blood samples were centrifuged at 2000× *g* for 10 min in a refrigerated centrifuge to separate plasma from the cellular components and stored at −70 °C until use.

### 4.4. Oral Glucose Tolerance Test (OGTT)

At 30 and 60 days of follow-up, and following an overnight fast, all the animals underwent an oral glucose tolerance test. Before the glucose overload, blood glucose concentration was measured using a FreeStyle Optium Neo glucometer (Abbott, Alameda, CA, USA), and a drop of blood was obtained from the tail vein after aseptic preparation. Subsequently, an oral glucose bolus (3 g/kg of body weight) was administered. Blood glucose concentrations were determined at 0, 30, 60, 90, 120, and 150, minutes in all experimental groups. The data were calculated and expressed as the AUC in mg/dL/h as previously described [68].

### 4.5. Determination of Lipid Profile in Plasma

Plasma concentrations of triglycerides (TGs), total cholesterol (TC), and high-density lipoprotein-cholesterol (HDL-c), were determined using commercial kits from BioAssay Systems (ETGA-200) (BioAssay Systems, Hayward, CA, USA) and Spinreact (Girona, Spain) (Cholesterol-LQ REF. 41020; HDL-c REF. 1001097). All parameters were processed according to the manufacturer’s instructions and read using a Multimode Reader (Sinergy H1, Biotek-Agilent, Santa Clara, CA, USA).

### 4.6. Markers of Liver Function in Plasma

Plasma concentrations of the hepatic enzymes alanine transaminase (ALT), aspartate transaminase (AST), ϒ-glutamyl transferase (GGT), and alkaline phosphatase (ALP) were measured using kits from Spinreact following the manufacturer’s instructions (ALT REF. 1001171; AST REF. 1001161; GGT REF. 1001186; ALP REF. 1001131). Total and direct bilirubin were determined with the Total Bilirubin-SL-X Reagent and Bilirubin Direct SL (SEKISUI Diagnostics, LLC, Burlington, MA, USA) according with the supplier instructions. All parameters were read using a Multimode Reader (Sinergy H1; Biotek-Agilent, Santa Clara, CA, USA).

### 4.7. Histological Analysis

#### 4.7.1. Hematoxylin/Eosin (H&E) Staining

Immediately after euthanasia, liver tissue slices were fixed by immersion in 10% formaldehyde in phosphate-buffered saline (PBS). Tissue slices, 1 mm in width, were de-hydrated and embedded in paraffin, sectioned at 4 µm, and stained with hematoxylin and eosin (H&E). For quantification, three animals were studied per time point [69] and experimental condition, a random count of 100 hepatic cells was performed and the percentage of hepatocytes with lipid cytoplasmic vacuoles distinctive of steatosis were determined. Data were expressed as percentages.

The presence of NASH was determined using the NAFLD activity scoring system (NAS), using the sum of three key histological components such as hepatocytes in ballooning degeneration, apoptosis, and lobular inflammation.

The NAS derives from the summation of individual scores for steatosis, lobular inflammation, and hepatocellular ballooning and ranges from 0 to 8 according to the score reported by Brunt E.M., and Tiniakos D.G., 2010 [27]. In the validation study, NAS of 1 or 2 corresponded to definitely not NASH, while a NAS score 5–8 correlated with definite NASH [44]. The degree of NASH activity and presence of fibrosis was determined according to the score reported by Brunt E.M., and Tiniakos D.G., 2010 [27].

#### 4.7.2. Red Oil Staining

Oil Red O staining was performed to assess lipid droplet formation in liver tissues following a previously described method [69]. Frozen liver tissues were embedded in Tissue-Tek^®^ O.C.T. compound for frozen sectioning using a cryostat. Tissue sections were cut into 6 µm slices and stained with Oil Red O for 20 min, followed by counterstaining with hematoxylin for 1 min. Histological analysis was conducted using a Leica light microscope. Five images were taken at random for each sample. Data were collected from at least three independent experiments.

#### 4.7.3. Assessment of the Liver Content of Triglycerides and Cholesterol

Triglycerides and cholesterol content were quantified in the liver tissue of all experimental groups. Briefly, 50 mg of tissue were homogenized in 300 µL of cold 50 mM phosphate-buffer saline, pH 7.4, and, subsequently, samples were centrifuged for 10 min at t 2050× *g* at 4 °C. The supernatant was separated and aliquoted. Samples to triglycerides content analysis were diluted five times and used to measure triglycerides with a commercial kit (BioAssay Systems (ETGA-200) after following the manufacturer’s instructions [70,71]. For the quantitation of cholesterol content in liver, 20 mg of liver was homogenized in 180 µL of cold ethanol, centrifuged at 1000× *g* for 10 min, and separated. The concentration was determined according to the supplier’s instructions [Total Cholesterol (TC) Colorimetric Assay Kit (Elabscience, Houston, TX, USA). The assays were read using a Multimode Reader (Sinergy H1; Biotek-Agilent, Santa Clara, CA, USA)].

### 4.8. Evaluation of Oxidative Stress

#### 4.8.1. Assessment of the Activity of the Mitochondrial Respiratory Complexes

Mitochondrial complex activities in liver tissue homogenates were assessed according to a previously described method [30]. First, after sacrifice, a sample of liver tissue (50 mg) was cooled by immersion in isolation buffer (225 mM D-mannitol, 75 mM sucrose, 1 mM EDTA, 5 mM HEPES, 0.1% BSA, pH 7.4) at 4 °C and then cut in small pieces. The tissue was homogenized in a glass Potter–Elvehjem with a Teflon VR pestle in isolation buffer and subsequently, mitochondria were obtained by differential centrifugation with percoll gradients [72]. The pellet was resuspended in 80 µL of BSA-free isolation buffer and the mitochondrial total protein was determined by the Bradford method.

Briefly, complex I (CI) activity was measured based on their capacity to oxidize NADH while reducing deubiquinone (DUB) to DubH2; DubH2 is then oxidized by 2,6-Dichlorophenolindophenol (DCPIP). CI activity is then determined by the disappearance of oxidized DCPIP at 600 nm and rotenone was used as their specific inhibitor. CII activity was measured in a separate assay using the succinate oxidation dependent CII capacity while reduce DUB to DUbH2. DUbH2 is then oxidized by DCPIP and the decrease in absorbance at 600 nm is proportional to CII activity. Maleic acid was added as specific inhibitor of CII. Absorbance was read at 37 °C using a BioTek Cytation 7 microplate reader (Agilent Instruments, Santa Clara, CA, USA). The specific activity of each complex was determined by subtracting the activity in the presence of the appropriate inhibitor from the non-inhibited activity. The results were expressed as nmol/min/mg protein, determined by the Bradford method [30].

#### 4.8.2. Activity of Antioxidant Enzymes in Liver

100 mg of liver tissue were homogenized in 50 mM PBS buffer, pH 7.4. To determine the activity of the endogenous antioxidant enzymes; superoxide dismutase (SOD), glutathione peroxidase (GPx), and glutathione reductase (GR), the supernatant was used according to previously described methods [30]. Briefly, SOD activity was determined spectrophotometrically at 560 nm using Nitro blue Tetrazolium Chloride (NBT) as the indicator reagent. One unit of SOD activity was defined as the amount of protein that inhibited 50% of NBT reduction, and the results were expressed as U/mg protein of total protein. GPx activity was determined by measuring the disappearance of β-nicotinamide-adenine dinucleotide phosphate reduced (NADPH) at 340 nm in a coupled reaction with GR and GPx. Units (U) were defined as the amount of enzyme that oxidizes one micromole NADPH/min, and data were expressed as U/mg total protein [30]. Changes in absorbance were measured at 37 °C using a BioTek Cytation 7 microplate reader (Agilent Instruments, Santa Clara, CA, USA).

#### 4.8.3. Determination of Concentrations of Malondialdehyde

Malondialdehyde (MDA) concentrations were determined as an index of lipid peroxidation in liver tissue homogenates, indirectly according to a published method. Samples were deproteinized with cold methanol (1:1 ratio), shaken vigorously for 1 min, and then 0.1 M sodium hydroxide (NaOH) and ammonium acetate were added before direct analysis. Analysis was performed by HPLC using a µBondapak C18 column (10 µm, 125% %Å, 3.9 × 150 mm) at a flow rate of 0.4 mL/min for 10 min at a wavelength of 200 nm. A standard curve (0 to 50 pmol/mL) was generated using the same method with MDA as standard. Calculations were performed by interpolation with the standard curve [73].

#### 4.8.4. Determination of Oxidized Proteins

Oxidized proteins were determined through the determination of carbonylated proteins in the liver tissue. 50 mg of liver tissue were processed to assess the carboxyl groups by measuring 2,4-dinitrophenylhydrazine (DNPH), which reacts to produce DNP-hydrazone, and it is quantified by spectrophotometry at 370 nm as previously described [30]. The data were expressed as mmoles of DNPH/mg of protein.

### 4.9. Analysis of Protein Expression of Nuclear Transcription Factors

To assess the expression of nuclear transcription factors, nuclear extracts were purified according to previously described methods [30]. Equal amounts of protein were loaded on SDS-PAGE gels in a minigel system (Mini-PROTEAN II, Bio-Rad, Hercules, CA, USA) and transferred to PVDF membranes (0.4 μm pore size, Merck-Millipore). The membranes were blocked with 5% milk in TBS-Tween for 1 h and were incubated overnight at 4 °C with the following antibodies: Nrf2 (Genetex, GTX 103322), ChREBP (Genetex, GTX 30677), and SREBP (Genetex, GTX79299). PCNA (Genetex, GTX100539) was used as a loading control. After incubation and washing, horseradish peroxidase (HRP)-conjugated antibodies were added. The proteins were detected by enhanced chemiluminescence (Clarity ECL Western Substrate, Bio-Rad, Hercules, CA, USA).

### 4.10. Statistical Analysis

All data are expressed as mean ± standard error of the mean (SEM). An unpaired *T*-test with Welch’s correction analysis was used to analyze the 30-day data. For the 60-day data, comparisons amongst groups were made using a one-way analysis of variance followed by Tukey’s test using GraphPad version 9.0.1 (GraphPad Software, La Jolla, CA, USA). Differences were considered significant when *p* < 0.05.

## 5. Conclusions

This study demonstrates that the empagliflozin/metformin combination effectively delays MASLD progression by improving liver and mitochondrial function and mitigating oxidative stress by enhancing endogenous antioxidant systems. These benefits were observed in both biochemical markers of liver damage and histological assessments, which revealed a reduction in hepatic lipid accumulation. Mechanistically, this appears to involve restoring key transcription factors governing lipid and carbohydrate metabolism, along with modulation of endogenous antioxidant enzymes. This co-therapy effectively controls MS-related risk factors, suggesting its potential to prevent MASLD development and its consideration as a therapeutic option for MS-induced MASLD. While both drugs individually exhibited positive effects, the combination demonstrated superior efficacy. These findings highlight the potential of this combination for both MASLD prophylaxis and the treatment of related metabolic diseases, warranting further investigation into these precise mechanisms of action and long-term effects, particularly regarding oxidative stress pathways and mitochondrial dynamics, to optimize its therapeutic application in metabolic syndrome.

## Figures and Tables

**Table 1:** Validation of the metabolic syndrome and plasma liver damage markers at 30 days of follow-up in all experimental groups, and before initiating pharmacological treatments.

		Western Diet and Sugary Drink			
Parameter	Control	MASLD	Empa/Met	Empa	Met
BW (g)	314.3 ± 12.71	344.7 ± 5.09 *	350.50 ± 9.86 *	335.5 ± 9.97	324.73 ± 5.59 *
FBG (mg/dL)	88.83 ± 2.10	102.3 ± 2.08 *	97.83 ± 2.71 *	106.8 ± 6.34 *	102.2 ± 3.09 *
TC (mg/dL)	59.62 ± 3.31	157.3 ± 6.75 *	176.9 ± 14.03 *	135.4 ± 9.60 *	163.4 ± 13.09 *
TG (mmol/L)	47.95 ± 5.69	166.2 ± 16.85 *	192.7 ± 28.59 *	152.1 ± 27.74 *	179.5 ± 48.39 *
HDL-c (mg/dL)	30.15 ± 1.58	20.99 ± 1.70 *	21.37 ± 3.64	23.07 ± 3.47 *	25.33 ± 4.18 *
OGTT [AUC (mg/dL/h)]	6630 ± 422.3	8512 ± 448.1 *	8349 ± 193.2 *	8676 ± 144.5 *	8481 ± 303.2 *
*ALT (U/L)*	8.47 ± 0.70	17.52 ± 1.96 *	14.79 ± 1.55 *	10.63 ± 1.58 *	10.85 ± 1.92 *
*AST (U/L)*	17.01 ± 0.99	29.16 ± 3.91 *	29.23 ± 3.37 *	28.74 ± 3.52 *	21.19 ± 2.85 *
*ALP (U/L)*	51.21 ± 5.45	124.5 ± 11.52 *	102.9 ± 4.44 *	112.4 ± 8.22 *	128.2 ± 13.18 *
*GGT (U/L)*	2.75 ± 0.21	7.65 ± 0.75 *	10.58 ± 1.36 *	7.67 ± 1.1 *	8.48 ± 1.12 *
*DB (mg/dL)*	0.77 ± 0.11	2.83 ± 0.46 *	3.06 ± 0.62 *	3.38 ± 0.53 *	2.85 ± 0.87 *
*TB (mg/dL)*	0.14 ± 0.007	1.15 ± 0.25 *	1.0 ± 0.26 *	1.15 ± 0.26 *	1.12 ± 0.20 *

Abbreviations: BW: body weight; FBG: fasting blood glucose; TC: total cholesterol; TG: triglycerides; HDL-c: high-density lipoprotein cholesterol; OGTT: oral glucose tolerance test; ALT: alanine transaminase; AST: aspartate aminotransferase; ALP: alkaline phosphatase; GGT: γ-Glutamyl transferase; DB: direct bilirubin; TB: total bilirubin. Empagliflozin (Empa), metformin (Met), empagliflozin + metformin (Empa + Met); metabolic dysfunction-associated steatotic liver disease (MASLD). Data are expressed as mean ± SEM, *n* = 6, and were analyzed by unpaired *t*-test with Welch’s correction. Statistical significance was established as * *p* < 0.05 vs. Control.

## Data Availability

The data presented in this study are available on request from the corresponding author.

## Supplementary Materials

The following supporting information can be downloaded at: https://www.mdpi.com/article/10.3390/ijms26189010/s1.

## Abbreviations

AST	Aspartate aminotransferase
ALT	Alanine transaminase
ALP	Alkaline phosphatase
AMPK	(AMP)-activated protein kinase alpha
BT	Bilirubin total
ChREBP	Carbohydrate-responsive element-binding protein
DB	Direct bilirubin
DNL	De novo lipogenesis
DNPH	2,4-dinitrophenylhydrazine
FFAs	Free fatty acids
FGB	Fasting blood glucose
GLP-1	Agonists, glucagon-like peptide 1
GGT	γ-Glutamyl transferase
GPx	Glutathione peroxidase
GR	Glutathione reductase
HDL-c	high-density lipoprotein-cholesterol
IL	Interleukin
IR	Insulin resistance
LXR	Liver X receptor alpha
MASLD	Metabolic dysfunction-associated steatotic liver disease
MS	Metabolic syndrome
MDA	Malondialdehyde
NAFLD	Non-alcoholic fatty liver disease
NASH	Non-alcoholic steatohepatitis
NLRP3	Nucleotide-binding domain, leucine-rich repeat-containing family, pyrin domain containing 3
Nrf2	Nuclear factor-erythroid 2 p45-related factor 2 (Nrf2)
OGTT	Oral glucose tolerance test
PPAR	Peroxisome proliferator-activated receptor
PUFAs	Polyunsaturated fatty acids
SBP	Systolic blood pressure
SGLT2i	Sodium-glucose cotransporter type 2 inhibitor
SREBP-1c	Sterol regulatory element binding protein-1c
SOD	Superoxide dismutase
TC	Total cholesterol
TG	Triglycerides
TNF-alpha	Tumour necrosis factor-alpha
T2DM	Type 2 diabetes mellitus
VLDL	Very low-density lipoproteins

## References

[1] Shaaban H.H., Alzaim I., El-Mallah A., Aly R.G., El-Yazbi A.F., Wahid A. (2022). Metformin, pioglitazone, dapagliflozin and their combinations ameliorate manifestations associated with NAFLD in rats via anti-inflammatory, anti-fibrotic, anti-oxidant and anti-apoptotic mechanisms. Life Sci..

[2] Yu L., Hong W., Lu S., Li Y., Guan Y., Weng X., Feng Z. (2022). The NLRP3 Inflammasome in Non-Alcoholic Fatty Liver Disease and Steatohepatitis: Therapeutic Targets and Treatment. Front. Pharmacol..

[3] Kuchay M.S., Krishan S., Mishra S.K., Farooqui K.J., Singh M.K., Wasir J.S., Bansal B., Kaur P., Jevalikar G., Gill H.K. (2018). Effect of Empagliflozin on Liver Fat in Patients with Type 2 Diabetes and Nonalcoholic Fatty Liver Disease: A Randomized Controlled Trial (E-LIFT Trial). Diabetes Care.

[4] Pouwels S., Sakran N., Graham Y., Leal A., Pintar T., Yang W., Kassir R., Singhal R., Mahawar K., Ramnarain D. (2022). Non-alcoholic fatty liver disease (NAFLD): A review of pathophysiology, clinical management and effects of weight loss. BMC Endocr. Disord..

[5] Katsiki N., Mikhailidis D.P., Mantzoros C.S. (2016). Non-alcoholic fatty liver disease and dyslipidemia: An update. Metabolism.

[6] Ratziu V., Boursier J., de Ledinghen V., Anty R., Costentin C., Bureau C. (2023). Confirmatory biomarker diagnostic studies are not needed when transitioning from NAFLD to MASLD. J. Hepatol..

[7] Chen L., Tao X., Zeng M., Mi Y., Xu L. (2023). Clinical and histological features under different nomenclatures of fatty liver disease: NAFLD, MAFLD, MASLD and MetALD. J. Hepatol..

[8] Wong R.J. (2024). Epidemiology of metabolic dysfunction-associated steatotic liver disease (MASLD) and alcohol-related liver disease (ALD). Metab. Target Organ. Damage.

[9] Younossi Z.M., Kalligeros M., Henry L. (2025). Epidemiology of metabolic dysfunction-associated steatotic liver disease. Clin. Mol. Hepatol..

[10] Remus P.A., Fratila O., Rus M., Anca C.A.R., Mihai V.C., Pantis C., Diaconu C.C., Bratu O., Bungau S., Nemeth S. (2020). Risk factors for adiposity in the urban population and influence on the prevalence of overweight and obesity. Exp. Ther. Med..

[11] Yin X., Guo X., Liu Z., Wang J. (2023). Advances in the Diagnosis and Treatment of Non-Alcoholic Fatty Liver Disease. Int. J. Mol. Sci..

[12] Diao L., Auger C., Konoeda H., Sadri A.-R., Amini-Nik S., Jeschke M.G. (2018). Hepatic steatosis associated with decreased β-oxidation and mitochondrial function contributes to cell damage in obese mice after thermal injury. Cell Death Dis..

[13] Radosavljevic T., Brankovic M., Samardzic J., Djuretić J., Vukicevic D., Vucevic D., Jakovljevic V. (2024). Altered Mitochondrial Function in MASLD: Key Features and Promising Therapeutic Approaches. Antioxidants.

[14] Muzurović E., Mikhailidis D.P., Mantzoros C. (2021). Non-alcoholic fatty liver disease, insulin resistance, metabolic syndrome and their association with vascular risk. Metabolism.

[15] Zhu Z., Zhang X., Pan Q., Zhang L., Chai J. (2023). In-depth analysis of de novo lipogenesis in non-alcoholic fatty liver disease: Mechanism and pharmacological interventions. Liver Res..

[16] Feng W., Bi Y., Li P., Yin T., Gao C., Shen S., Gao L., Yang D., Zhu D. (2018). Effects of liraglutide, metformin and gliclazide on body composition in patients with both type 2 diabetes and non-alcoholic fatty liver disease: A randomized trial. J. Diabetes Investig..

[17] Taheri H., Malek M., Ismail-Beigi F., Zamani F., Sohrabi M., Babaei M.R., Khamseh M.E. (2020). Effect of Empagliflozin on Liver Steatosis and Fibrosis in Patients with Non-Alcoholic Fatty Liver Disease Without Diabetes: A Randomized, Double-Blind, Placebo-Controlled Trial. Adv. Ther..

[18] Ratziu V., Francque S., Sanyal A. (2022). Breakthroughs in therapies for NASH and remaining challenges. J. Hepatol..

[19] Ntikoudi A., Papachristou A., Tsalkitzi A., Margari N., Evangelou E., Vlachou E. (2025). Metabolic-Associated Steatotic Liver Disease (MASLD) and Type 2 Diabetes: Mechanisms, Diagnostic Approaches, and Therapeutic Interventions. Diabetology.

[20] Doycheva I., Loomba R. (2014). Effect of Metformin on Ballooning Degeneration in Nonalcoholic Steatohepatitis (NASH): When to Use Metformin in Nonalcoholic Fatty Liver Disease (NAFLD). Adv. Ther..

[21] Viollet B., Guigas B., Sanz G.N., Leclerc J., Foretz M., Andreelli F. (2012). Cellular and molecular mechanisms of metformin: An overview. Clin. Sci..

[22] Gkiourtzis N., Michou P., Moutafi M., Glava A., Cheirakis K., Christakopoulos A., Vouksinou E., Fotoulaki M. (2023). The benefit of metformin in the treatment of pediatric non-alcoholic fatty liver disease: A systematic review and meta-analysis of randomized controlled trials. Eur. J. Pediatr..

[23] Nasiri-Ansari N., Nikolopoulou C., Papoutsi K., Kyrou I., Mantzoros C.S., Kyriakopoulos G., Chatzigeorgiou A., Kalotychou V., Randeva M.S., Chatha K. (2021). Empagliflozin Attenuates Non-Alcoholic Fatty Liver Disease (NAFLD) in High Fat Diet Fed ApoE^(-/-)^ Mice by Activating Autophagy and Reducing ER Stress and Apoptosis. Int. J. Mol. Sci..

[24] Bica I.-C., Stoica R.A., Salmen T., Janež A., Volčanšek Š., Popovic D., Muzurovic E., Rizzo M., Stoian A.P. (2023). The Effects of Sodium-Glucose Cotransporter 2-Inhibitors on Steatosis and Fibrosis in Patients with Non-Alcoholic Fatty Liver Disease or Steatohepatitis and Type 2 Diabetes: A Systematic Review of Randomized Controlled Trials. Medicina.

[25] Niu S., Ren Q., Chen S., Pan X., Yue L., Chen X., Li Z., Zhen R. (2023). Metabolic and Hepatic Effects of Empagliflozin on Nonalcoholic Fatty Liver Mice. Diabetes Metab. Syndr. Obes..

[26] Friedman S.L., Neuschwander-Tetri B.A., Rinella M., Sanyal A.J. (2018). Mechanisms of NAFLD development and therapeutic strategies. Nat. Med..

[27] Brunt E.M., Tiniakos D.G. (2010). Histopathology of nonalcoholic fatty liver disease. World J. Gastroenterol..

[28] García-Arroyo F.E., Gonzaga-Sánchez G., Tapia E., Muñoz-Jiménez I., Manterola-Romero L., Osorio-Alonso H., Arellano-Buendía A.S., Pedraza-Chaverri J., Roncal-Jiménez C.A., Lanaspa M.A. (2021). Osthol Ameliorates Kidney Damage and Metabolic Syndrome Induced by a High-Fat/High-Sugar Diet. Int. J. Mol. Sci..

[29] García-Arroyo F.E., Gonzaga-Sánchez G., Silva-Palacios A., Roldán F.J., Loredo-Mendoza M.L., Alvarez-Alvarez Y.Q., Coyotl J.A.d.L.S., Orozco K.A.V., Tapia E., Osorio-Alonso H. (2023). Osthole Prevents Heart Damage Induced by Diet-Induced Metabolic Syndrome: Role of Fructokinase (KHK). Antioxidants.

[30] Buendia A.S.A., Rojas J.G.J., García-Arroyo F., Trejo O.E.A., Sánchez-Muñoz F., Argüello-García R., Sánchez-Lozada L.G., Bojalil R., Osorio-Alonso H. (2023). Antioxidant and anti-inflammatory effects of allicin in the kidney of an experimental model of metabolic syndrome. PeerJ.

[31] Simoes I.C.M., Karkucinska-Wieckowska A., Janikiewicz J., Szymanska S., Pronicki M., Dobrzyn P., Dabrowski M., Dobrzyn A., Oliveira P.J., Zischka H. (2020). Western Diet Causes Obesity-Induced Nonalcoholic Fatty Liver Disease Development by Differentially Compromising the Autophagic Response. Antioxidants.

[32] Makri E.S., Makri E., Goulas A., Xanthopoulos K., Polyzos S.A. (2024). Animal studies of sodium-glucose co-transporter 2 inhibitors in nonalcoholic fatty liver disease. Ann. Gastroenterol..

[33] Bence K.K., Birnbaum M.J. (2021). Metabolic drivers of non-alcoholic fatty liver disease. Mol. Metab..

[34] Jensen T., Abdelmalek M.F., Sullivan S., Nadeau K.J., Green M., Roncal C., Nakagawa T., Kuwabara M., Sato Y., Kang D.-H. (2018). Fructose and sugar: A major mediator of non-alcoholic fatty liver disease. J. Hepatol..

[35] Caturano A., Galiero R., Loffredo G., Vetrano E., Medicamento G., Acierno C., Rinaldi L., Marrone A., Salvatore T., Monda M. (2023). Effects of a Combination of Empagliflozin Plus Metformin vs. Metformin Monotherapy on NAFLD Progression in Type 2 Diabetes: The IMAGIN Pilot Study. Biomedicines.

[36] Tahara A., Takasu T. (2020). Therapeutic Effects of SGLT2 Inhibitor Ipragliflozin and Metformin on NASH in Type 2 Diabetic Mice. Endocr. Res..

[37] Esmaeili A., Azar R.P., Hosseiniazar M.M., Gharabagh L.H. (2024). Empagliflozin add-on therapy is superior to metformin monotherapy in diabetic patients with NAFLD: An open-label, single-center, pilot clinical trial. J. Gen. Fam. Med..

[38] Lai L.-L., Vethakkan S.R., Mustapha N.R.N., Mahadeva S., Chan W.-K. (2019). Empagliflozin for the Treatment of Nonalcoholic Steatohepatitis in Patients with Type 2 Diabetes Mellitus. Dig. Dis. Sci..

[39] Romera I., Ampudia-Blasco F.J., Pérez A., Ariño B., Pfarr E., Kis S.G., Naderali E. (2016). Efficacy and safety of empagliflozin in combination with other oral hypoglycemic agents in patients with type 2 diabetes mellitus. Endocrinol. Nutr. Eng. Ed..

[40] Smirne C., Croce E., Di Benedetto D., Cantaluppi V., Comi C., Sainaghi P.P., Minisini R., Grossini E., Pirisi M. (2022). Oxidative Stress in Non-Alcoholic Fatty Liver Disease. Livers.

[41] Wang Y., Ding Y., Sun P., Zhang W., Xin Q., Wang N., Niu Y., Chen Y., Luo J., Lu J. (2022). Empagliflozin-Enhanced Antioxidant Defense Attenuates Lipotoxicity and Protects Hepatocytes by Promoting FoxO3a- and Nrf2-Mediated Nuclear Translocation via the CAMKK2/AMPK Pathway. Antioxidants.

[42] Smith B.K., Marcinko K., Desjardins E.M., Lally J.S., Ford R.J., Steinberg G.R. (2016). Treatment of nonalcoholic fatty liver disease: Role of AMPK. Am. J. Physiol. Metab..

[43] Arrese M., Cabrera D., Kalergis A.M., Feldstein A.E. (2016). Innate Immunity and Inflammation in NAFLD/NASH. Dig. Dis. Sci..

[44] Takahashi Y., Fukusato T. (2014). Histopathology of nonalcoholic fatty liver disease/nonalcoholic steatohepatitis. World J. Gastroenterol..

[45] Harrison S.A., Bedossa P., Guy C.D., Schattenberg J.M., Loomba R., Taub R., Labriola D., Moussa S.E., Neff G.W., Rinella M.E. (2024). A Phase 3, Randomized, Controlled Trial of Resmetirom in NASH with Liver Fibrosis. N. Engl. J. Med..

[46] Harrison S.A., Wong V.W.-S., Okanoue T., Bzowej N., Vuppalanchi R., Younes Z., Kohli A., Sarin S., Caldwell S.H., Alkhouri N. (2020). Selonsertib for patients with bridging fibrosis or compensated cirrhosis due to NASH: Results from randomized phase III STELLAR trials. J. Hepatol..

[47] Albhaisi S., Noureddin M. (2021). Current and Potential Therapies Targeting Inflammation in NASH. Front. Endocrinol..

[48] Janić M., Janež A., Šabović M., El-Tanani M., Rangraze I., Rizzo M., Lunder M. (2024). Glucometabolic Efficacy of the Empagliflozin/Metformin Combination in People with Type 1 Diabetes and Increased Cardiovascular Risk: A Sub-Analysis of a Pilot Randomized Controlled Trial. J. Clin. Med..

[49] Janić M., Cankar M., Šmid J., Štiglic A.F., Jerin A., Šabovič M., Janež A., Lunder M., Sasso F.C. (2022). Empagliflozin-Metformin Combination Has Antioxidative and Anti-Inflammatory Properties that Correlate with Vascular Protection in Adults with Type 1 Diabetes. J. Diabetes Res..

[50] Shi M., Zhang H., Wang W., Zhang X., Liu J., Wang Q., Wang Y., Zhang C., Guo X., Qiao Q. (2023). Effect of dapagliflozin on liver and pancreatic fat in patients with type 2 diabetes and non-alcoholic fatty liver disease. J. Diabetes Its Complicat..

[51] Milder J.B., Liang L.-P., Patel M. (2010). Acute oxidative stress and systemic Nrf2 activation by the ketogenic diet. Neurobiol. Dis..

[52] Na Kim M., Moon J.H., Cho Y.M. (2021). Sodium-glucose cotransporter-2 inhibition reduces cellular senescence in the diabetic kidney by promoting ketone body-induced NRF2 activation. Diabetes Obes. Metab..

[53] Chen Y., Lin Y., Lin J., Yang N., Chen M. (2018). Sugary Kefir Strain *Lactobacillus mali* APS1 Ameliorated Hepatic Steatosis by Regulation of SIRT-1/Nrf-2 and Gut Microbiota in Rats. Mol. Nutr. Food Res..

[54] Chowdhry S., Nazmy M.H., Meakin P.J., Dinkova-Kostova A.T., Walsh S.V., Tsujita T., Dillon J.F., Ashford M.L., Hayes J.D. (2010). Loss of Nrf2 markedly exacerbates nonalcoholic steatohepatitis. Free Radic. Biol. Med..

[55] Leng W., Wu M., Pan H., Lei X., Chen L., Wu Q., Ouyang X., Liang Z. (2019). The SGLT2 inhibitor dapagliflozin attenuates the activity of ROS-NLRP3 inflammasome axis in steatohepatitis with diabetes mellitus. Ann. Transl. Med..

[56] Xu C., Wang W., Zhong J., Lei F., Xu N., Zhang Y., Xie W. (2018). Canagliflozin exerts anti-inflammatory effects by inhibiting intracellular glucose metabolism and promoting autophagy in immune cells. Biochem. Pharmacol..

[57] Qiao P., Jia Y., Ma A., He J., Shao C., Li X., Wang S., Yang B., Zhou H. (2022). Dapagliflozin protects against nonalcoholic steatohepatitis in db/db mice. Front. Pharmacol..

[58] Haukeland J.W., Konopski Z., Eggesbø H.B., von Volkmann H.L., Raschpichler G., Bjøro K., Haaland T., Løberg E.M., Birkeland K. (2009). Metformin in patients with non-alcoholic fatty liver disease: A randomized, controlled trial. Scand. J. Gastroenterol..

[59] Jalali M., Rahimlou M., Mahmoodi M., Moosavian S.P., Symonds M.E., Jalali R., Zare M., Imanieh M.H., Stasi C. (2020). The effects of metformin administration on liver enzymes and body composition in non-diabetic patients with non-alcoholic fatty liver disease and/or non-alcoholic steatohepatitis: An up-to date systematic review and meta-analysis of randomized controlled trials. Pharmacol. Res..

[60] Fullerton M.D., Galic S., Marcinko K., Sikkema S., Pulinilkunnil T., Chen Z.-P., O’NEill H.M., Ford R.J., Palanivel R., O’BRien M. (2013). Single phosphorylation sites in Acc1 and Acc2 regulate lipid homeostasis and the insulin-sensitizing effects of metformin. Nat. Med..

[61] Park J., Rah S.-Y., An H.S., Lee J.Y., Roh G.S., Ryter S.W., Park J.W., Yang C.H., Surh Y.-J., Kim U.-H. (2023). Metformin-induced TTP mediates communication between Kupffer cells and hepatocytes to alleviate hepatic steatosis by regulating lipophagy and necroptosis. Metabolism.

[62] Yap F., Craddock L., Yang J. (2011). Mechanism of AMPK suppression of LXR-dependent Srebp-1c transcription. Int. J. Biol. Sci..

[63] Lin M.J., Dai W., Scott M.J., Li R., Zhang Y.Q., Yang Y., Chen L.Z., Huang X.S. (2017). Metformin improves nonalcoholic fatty liver disease in obese mice via down-regulation of apolipoprotein A5 as part of the AMPK/LXRα signaling pathway. Oncotarget.

[64] Abdelhamid A.M., Youssef M.E., El-Fattah E.E.A., Gobba N.A., Gaafar A.G.A., Girgis S., Shata A., Hafez A.-M., El-Ahwany E., Amin N.A. (2021). Blunting p38 MAPKα and ERK1/2 activities by empagliflozin enhances the antifibrotic effect of metformin and augments its AMPK-induced NF-κB inactivation in mice intoxicated with carbon tetrachloride. Life Sci..

[65] Arefin A., Gage M.C. (2023). Metformin, Empagliflozin, and Their Combination Modulate Ex-Vivo Macrophage Inflammatory Gene Expression. Int. J. Mol. Sci..

[66] Rong L., Zou J., Ran W., Qi X., Chen Y., Cui H., Guo J. (2023). Advancements in the treatment of non-alcoholic fatty liver disease (NAFLD). Front. Endocrinol..

[67] Mascolo A., Di Napoli R., Balzano N., Cappetta D., Urbanek K., De Angelis A., Scisciola L., Di Meo I., Sullo M.G., Rafaniello C. (2022). Safety profile of sodium glucose co-transporter 2 (SGLT2) inhibitors: A brief summary. Front. Cardiovasc. Med..

[68] Sakaguchi K., Takeda K., Maeda M., Ogawa W., Sato T., Okada S., Ohnishi Y., Nakajima H., Kashiwagi A. (2015). Glucose area under the curve during oral glucose tolerance test as an index of glucose intolerance. Diabetol. Int..

[69] Long J.-K., Dai W., Zheng Y.-W., Zhao S.-P. (2019). miR-122 promotes hepatic lipogenesis via inhibiting the LKB1/AMPK pathway by targeting Sirt1 in non-alcoholic fatty liver disease. Mol. Med..

[70] Lifante J., de la Fuente-Fernández M., Román-Carmena M., Fernandez N., García D.J., Granado M., Ximendes E. (2022). In vivo grading of lipids in fatty liver by near-infrared autofluorescence and reflectance. J. Biophotonics.

[71] Spitler K.M., Shetty S.K., Cushing E.M., Sylvers-Davie K.L., Davies B.S.J. (2021). Regulation of plasma triglyceride partitioning by adipose-derived ANGPTL4 in mice. Sci. Rep..

[72] Granados-Castro L.F., Rodríguez-Rangel D.S., Montaño M., Ramos C., Pedraza-Chaverri J. (2013). Wood smoke exposure induces a decrease in respiration parameters and in the activity of respiratory complexes I and IV in lung mitochondria from guinea pigs. Environ. Toxicol..

[73] Varela-López E., del Valle-Mondragón L., Castrejón-Téllez V., Pérez-Torres I., Arenas A.P., Rojas F.M., Guarner-Lans V., Vargas-González A., Pastelín-Hernández G., Torres-Narváez J.C. (2021). Role of the Transient Receptor Potential Vanilloid Type 1 (TRPV1) in the Regulation of Nitric Oxide Release in Wistar Rat Aorta. Oxidative Med. Cell. Longev..

