# Supplementary material for: The Combination Empagliflozin/Metformin Attenuates the Progression of Metabolic Dysfunction-Associated Steatotic Liver Disease in a Diet-Induced Experimental Rat Model
Source: Int J Mol Sci. 2025 Sep 16;26(18):9010. doi: 10.3390/ijms26189010 (PMC12470015; doi:10.3390/ijms26189010)
Supplement: Supplementary file 1 [file ijms-26-09010-s001.zip › ijms-3792770-supplementary.pdf]

Table S1. Composition of maintenance and western type diets.

Diet	Composition (%)	Calories provided (%)
Maintenance	LabDiet® 5001	Protein 28.66<br>Fat 13.11<br>Carbohydrate 58.22
Western-type	1% Cholesterol<br>0.5% Cholic acid<br>5% Butter<br>30% Powdered sugar<br>10% Casein<br>2% NaCl<br>51.5% Regular diet	Protein 23.10<br>Fat 20.93<br>Carbohydrate 56.46
Sugary drink (11%)	Glucose:fructose (3.8:7.2)	Carbohydrate 44

Supplementary figures

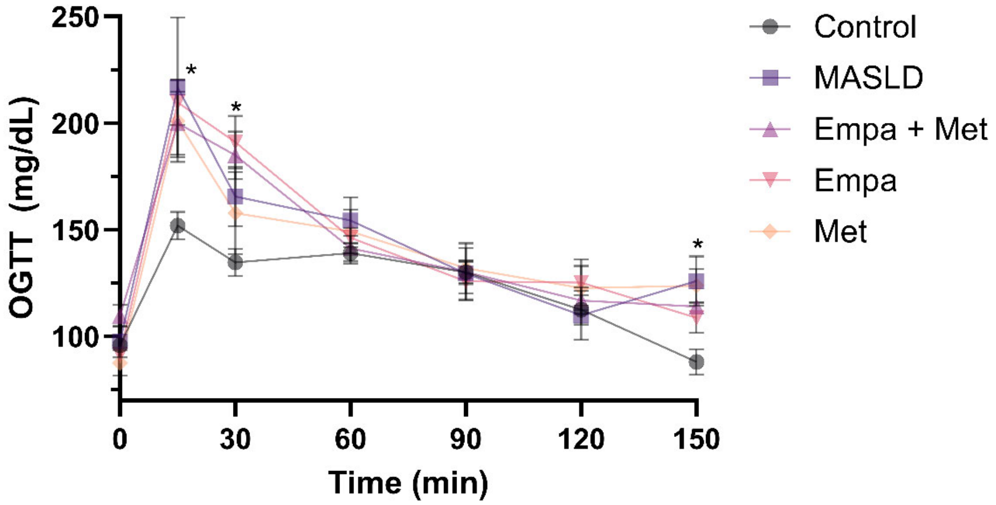

Figure S1. Oral glucose tolerance test at 30 days of follow-up, and before starting the treatments with Empagliflozin (Empa), Metformin (Met), the combination empagliflozin metformin and the Metabolic Dysfunction-Associated Steatotic Liver Disease (MASLD) group. Data are expressed as mean ± SEM, n=6, analyzed by one-way ANOVA. Statistical significance was established as *P< 0.05 vs. Control.

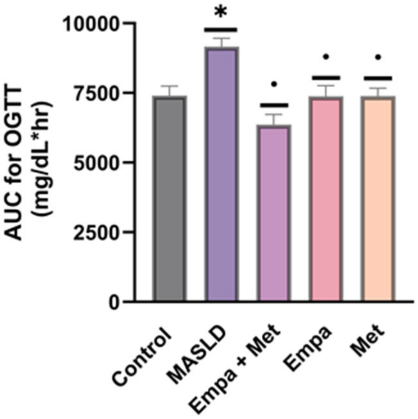

Figure S2. Oral glucose tolerance test (Area under curve) at 60 days of total follow-up, and after of 30 days of treatments with Empagliflozin (Empa), Metformin (Met), the combination empagliflozin metformin (Empa+Met), and the Metabolic Dysfunction-Associated Steatotic Liver Disease (MASLD) group. Data are expressed as mean ± SEM, n=6, analyzed by one-way ANOVA. Statistical significance was established as *P< 0.05 vs. Control; •P< 0.05 vs. MASLD

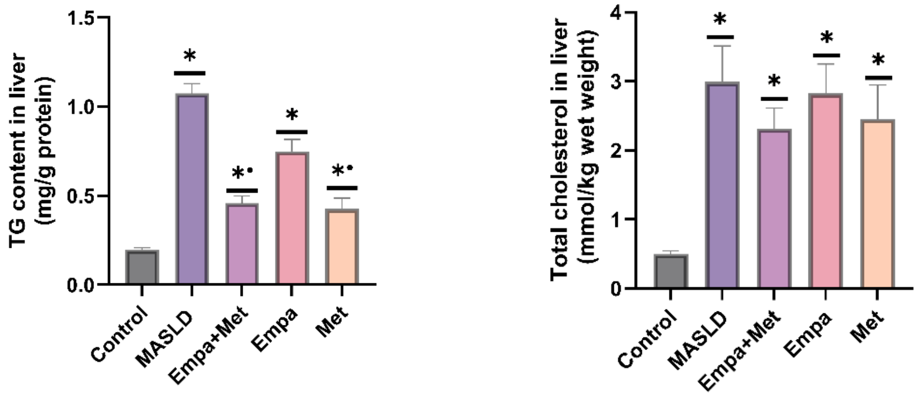

(**a**) (**b**)

Figure S3. Effect of the treatments on the liver content of (a) Triglycerides and (b) Total Cholesterol after 30 days of treatment. Empagliflozin (Empa), Metformin (Met), the combination empagliflozin + Metformin (Empa+Met), and Metabolic Dysfunction-Associated Steatotic Liver Disease (MASLD). Data are expressed as mean ± SEM, n=6, analyzed by one-way ANOVA. Statistical significance was established as *P<0.05 vs. Control; •P< 0.05 vs. MASLD.
